# How can we predict mortality in acute stroke?

**DOI:** 10.1590/1806-9282.20250057

**Published:** 2025-06-16

**Authors:** Egidio Bezerra da Silva, Williames Matheus Malaquias da Silva, Wendel Aguiar Carlini, Luana Karoline Castro Silva, Guilherme Dias Miranda Salgado Ribeiro, Julio Martinez Santos, Johnnatas Mikael Lopes

**Affiliations:** 1 Universidade Federal do Vale do São Francisco – Paulo Afonso (BA), Brazil.; 2 Universidade Federal do Ceará – Fortaleza (CE), Brazil.

Dear Editor,

Stroke has the second highest mortality rate in the world and is estimated to remain in this position until 2030^1^. Studies differ little on stroke mortality rates, ranging from 7.13 to 11.7% during hospitalization, up to 19% after 1 month, and 22% within 1 year after stroke. The overall mortality has been related to a history of previous stroke, greater stroke severity (National Institutes of Health Stroke Scale [NIHSS] ≥14), and cardiovascular and respiratory complications. In addition, low socioeconomic status, decreased level of consciousness, age over 64 years, atrial fibrillation (AF), coronary heart disease, hypertension, and need for surgery are also indicated as predictors of long-term mortality. The main predictors of mortality within 1 year have been dependence on activities of daily living and the presence of comorbidities^2,3^.

The clinical presentation of stroke is quite varied, but in the vast majority of cases, it involves the sudden onset of some focal clinical deficit, related to the specific site of the central nervous system where the ischemic process has occurred. However, the main symptoms that can be found during a stroke are severe headache, sudden and acute loss of balance and coordination, hemispatial inattention, blurred or double vision, homonymous hemianopsia, hemiparesis, hemianesthesia, facial asymmetry and flaccidity of the hemilateral facial muscles, and aphasia^4,5^.

Observing the research entitled "The role of the prognostic nutritional index in predicting mortality in stroke patients"^6^, published in this journal, a possible analytical bias is identified, which needs to be clarified with a new analysis in order not to compromise clinical practice in cerebrovascular emergency and urgency services. The data presented show that, just as there is a statistically significant distinction regarding the nutritional prognostic index (PNI) between those who survived and those who died, there is also the presence of AF and blood markers, such as albumin and leukocyte count. However, only the receiver operating characteristic (ROC) curve for PNI was presented. The question that remains is: Is PNI biased by the imbalance of AF cases and blood markers?

The results presented in Table 3 of the study do not allow us to resolve this crucial doubt since only a stratified analysis of the data by the AF variables and blood markers with the respective ROC curves would allow us to verify this bias^7^. Another alternative would be to construct a generalizable linear model with Poisson distribution to control for confounding biases between the possible predictors as well as to identify interactions between them, which would assist in the construction of specific strata for the elaboration of the ROC curve^8^. This analytical approach would eliminate the doubt about the origin of the prediction of in-hospital mortality in cases of acute stroke for the variables presented.
